# Intermittent hypoxia-induced insulin resistance is associated with alterations in white fat distribution

**DOI:** 10.1038/s41598-017-11782-0

**Published:** 2017-09-11

**Authors:** Laureline Poulain, Hervé Mathieu, Amandine Thomas, Anne-Laure Borel, Chantal Remy, Patrick Levy, Claire Arnaud, Maurice Dematteis

**Affiliations:** 1 Université Grenoble Alpes, Laboratoire HP2, Grenoble, F-38042 France; 2 Inserm U1042, Laboratoire HP2, Grenoble, F-38042 France; 3 Université Grenoble Alpes, IRMaGe, Grenoble, F-38000 France; 4 Inserm, US017, Grenoble, F-38043 France; 5 CNRS, UMS 3552, Grenoble, F-38043 France; 6 CHU Grenoble-Alpes, IRMaGe, Grenoble, F-38000 France; 7 Inserm, U1216, Grenoble, F-38000 France; 8 CHU Grenoble-Alpes, Laboratoires du Sommeil et EFCR, Grenoble, F-38043 France; 9 CHU Grenoble-Alpes, Service d’Addictologie, Grenoble, F-38043 France

## Abstract

Sleep apnea syndrome is characterized by repetitive upper airway collapses during night leading to intermittent hypoxia (IH). The latter is responsible for metabolic disturbances that rely, at least in part, on abdominal white fat inflammation. Besides qualitative alterations, we hypothesized that IH could also modify body fat distribution, a key factor for metabolic complications. C57BL6 mice exposed to IH (21–5% FiO_2_, 60 s cycle, 8 h/day) or air for 6 weeks were investigated for topographic fat alterations (whole-body MRI). Specific role of epididymal fat in IH-induced metabolic dysfunctions was assessed in lipectomized or sham-operated mice exposed to IH or air. Whereas total white fat volume was unchanged, IH induced epididymal adipose tissue (AT) loss with non-significant increase in subcutaneous and mesenteric fat. This was associated with impaired insulin sensitivity and secretion. Epididymal lipectomy led to increased subcutaneous fat in the perineal compartment and prevented IH-induced metabolic disturbances. IH led to reduced epididymal AT and impaired glucose regulation. This suggests that, rather than epididymal AT volume, qualitative fat alterations (i.e. inflammation) could represent the main determinant of metabolic dysfunction. This deterioration of glucose regulation was prevented in epididymal-lipectomized mice, possibly through prevention of IH-induced epididymal AT alterations and compensatory increase in subcutaneous AT.

## Introduction

Obstructive sleep apnea (OSA) is a worldwide public-health problem affecting at least 10% of the middle aged population and representing a main cause of cardiometabolic morbidity and mortality^1^. OSA is characterized by repetitive upper airway collapses resulting in intermittent hypoxia (IH), exaggerated negative intrathoracic pressure and sleep fragmentation. Experimental models of OSA allowed electing intermittent hypoxia (IH) as the most detrimental OSA-parameter for cardiometabolic complications^2^. Obesity is a major confounding factor as it is frequently associated with OSA and causes similar detrimental consequences than OSA^3^. Indeed, OSA and obesity share common pathophysiological pathways, including increased sympathetic activity, oxidative stress and chronic low-grade inflammation, contributing to metabolic alterations such as insulin resistance^4^. Obesity is a well-known independent risk factor for diabetes and cardiovascular events, and is directly associated with increased mortality^5^. However, rather than obesity *per se*, there are growing evidence that the pattern of fat distribution within the body is a major determinant of the metabolic profile and the severity of complications^6^. The abdominal fat is indeed composed of two major fat compartments: the subcutaneous and the intra-abdominal fat, the latter including the intraperitoneal (also called visceral) omental and mesenteric fat, and the retroperitoneal perirenal fat. It is well known that excessive visceral fat in a subject with normal body mass index (BMI) is accompanied by a greater risk of developing metabolic syndrome compared to a subject with higher BMI and presenting lesser amount of visceral fat^7^. The excessive visceral white adipose tissue (WAT) releases numerous cytokines and adipokines with autocrine, paracrine and endocrine signaling that participate to metabolic dysregulations, as well as vascular alterations and chronic inflammation^8^. We and others have recently shown that IH exerts detrimental effects on the epididymal WAT of non-obese mice, characterized by structural and inflammatory remodeling^9–11^ as well as alterations of the lipoprotein clearance pathway^12, 13^. These WAT alterations contribute to IH-associated cardiovascular complications as epididymal lipectomy before IH exposure partially attenuated IH-induced dyslipidemia and atherogenesis^9^. As mentioned above, the pattern of fat distribution could also be a good determinant of the metabolic status and the severity of cardiovascular complications^8^. As we previously observed smaller epididymal fat pads but similar body weight in mice exposed to IH compared to normoxic animals^9^, we suggest that IH, in addition to structural and inflammatory WAT remodeling, could induce fat redistribution that could participate to cardiometabolic disturbances. Therefore the aims of the present study were:

1. to investigate the effects of IH on regional distribution of body fat using magnetic resonance imaging in a non-obese mouse model;
2. to assess whether the IH-related pattern of fat distribution was associated with metabolic alterations;
3. to evaluate whether epididymal lipectomy before IH exposure had a protective role on IH-induced metabolic alterations and influenced the IH-related pattern of fat distribution.

## Results

### IH induces alterations of body fat distribution

**Figure 1:**
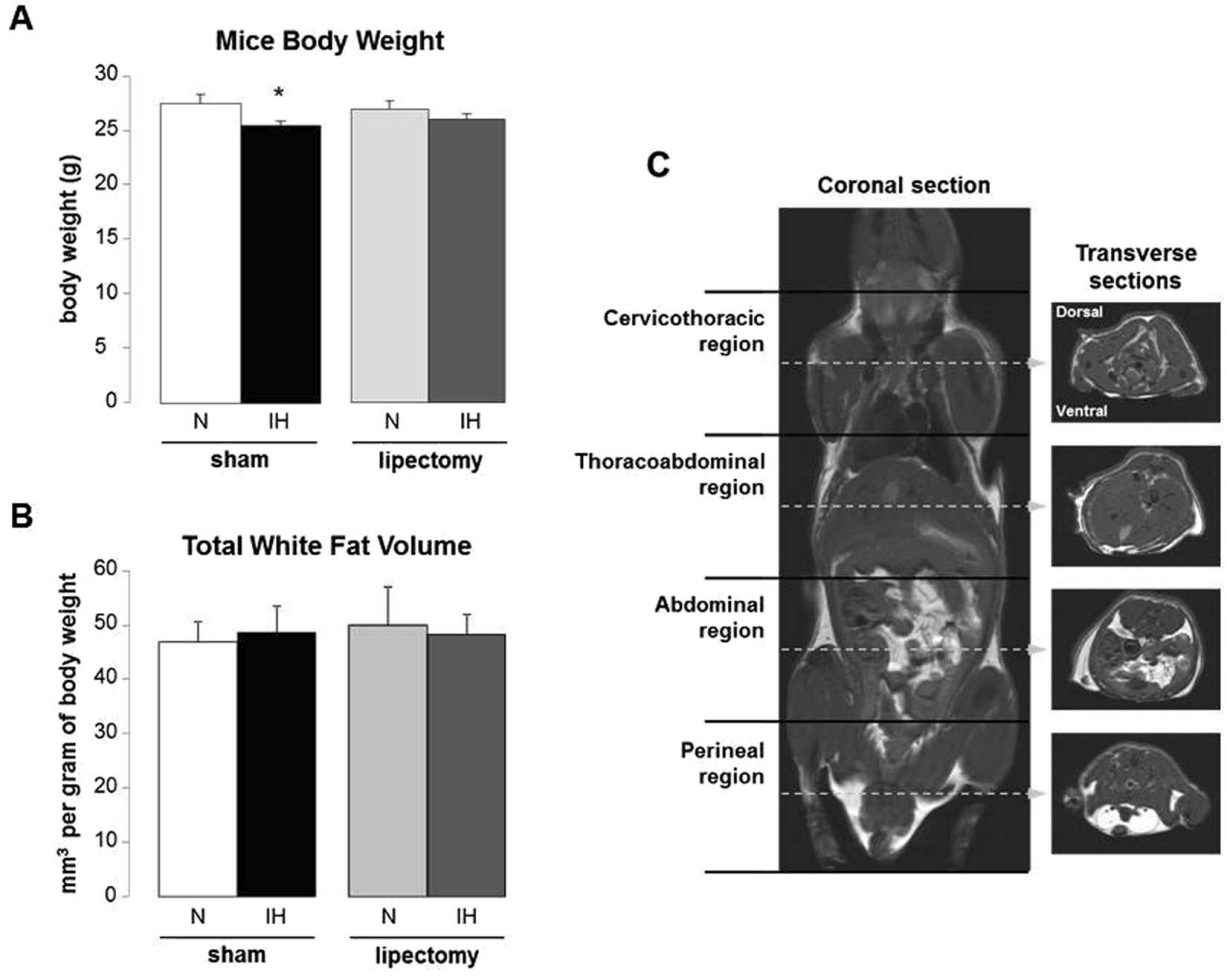
MRI quantification of total white adipose tissue volume in mice exposed to intermittent hypoxia or normoxia. (**A**) Mice body weight after 6 weeks of intermittent hypoxia (IH) or air (N) in lipectomized and sham-operated C57BL6 mice, n = 6–7 per group. (**B**) Total white adipose tissue volume after 6 weeks of IH or N in lipectomized and sham-operated C57BL6 mice, n = 4 per group. (**C**) Representative coronal and tranverse T1-weighted images.

**Figure 2:**
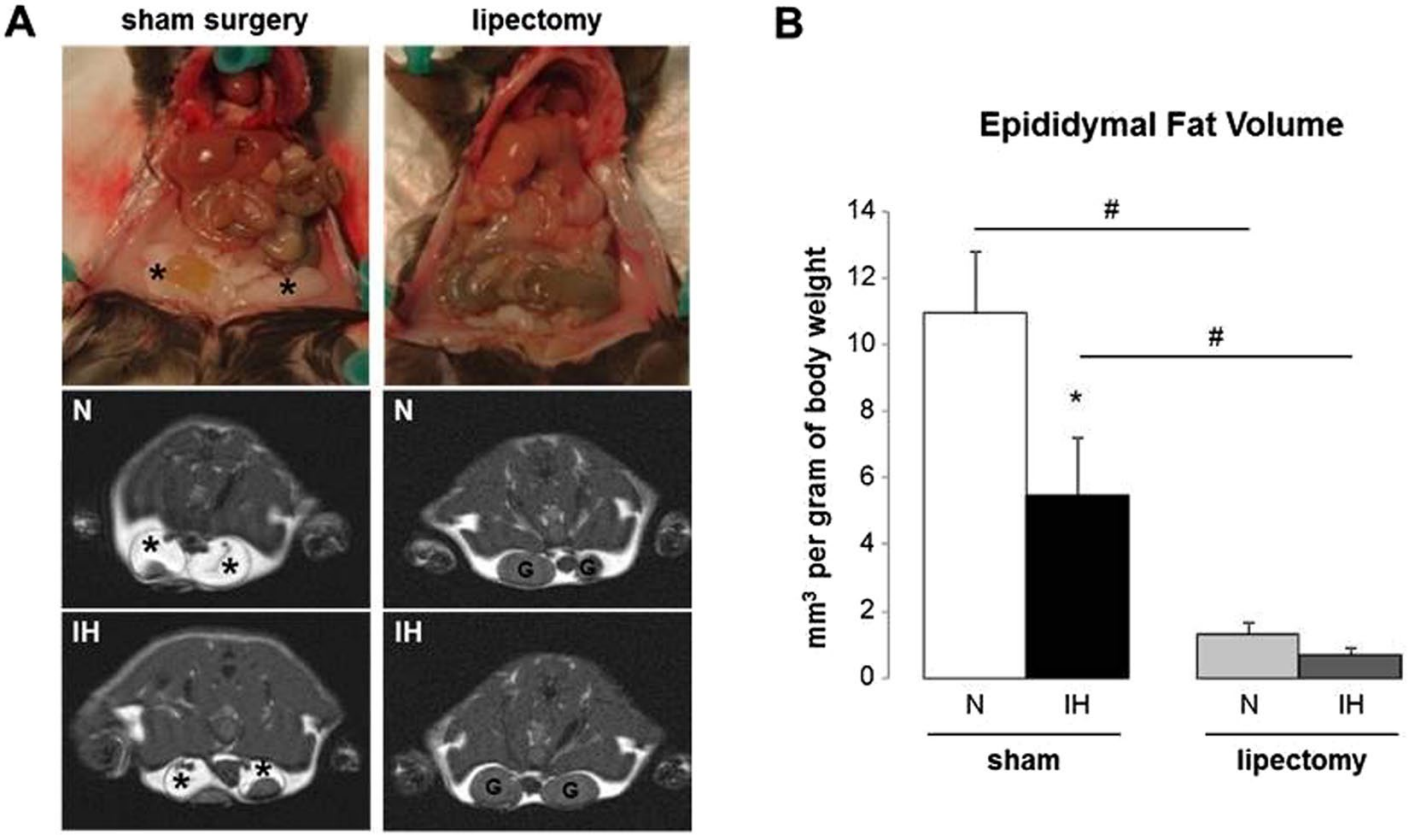
MRI quantification of epididymal white adipose tissue volume in mice exposed to intermittent hypoxia or normoxia. (**A**) Representative photographs and transverse MRI slices of epididymal fat pads (arrow) and gonads (G). (**B**) Epididymal fat volume after 6 weeks of intermittent hypoxia (IH) or air (N) in lipectomized and sham-operated C57BL6 mice, n = 4 per group; *p < 0.05 N-sham *vs* IH-sham, ^#^p < 0.05 N-sham *vs* N-lipectomy and IH-sham *vs* IH-lipectomy.

**Figure 3:**
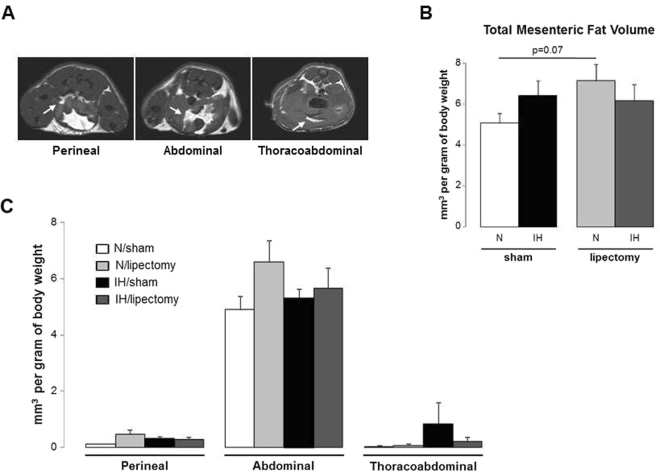
MRI quantification of mesenteric white adipose tissue volume in mice exposed to intermittent hypoxia or normoxia. (**A**) Representative transverse MRI slices (arrow showing the mesenteric fat), (**B**) total and (**C**) regional mesenteric fat volume in the 3 parts of the abdominal cavity after 6 weeks of intermittent hypoxia (IH) or air (N) in lipectomized or sham-operated mice, n = 4 per group.

**Figure 4:**
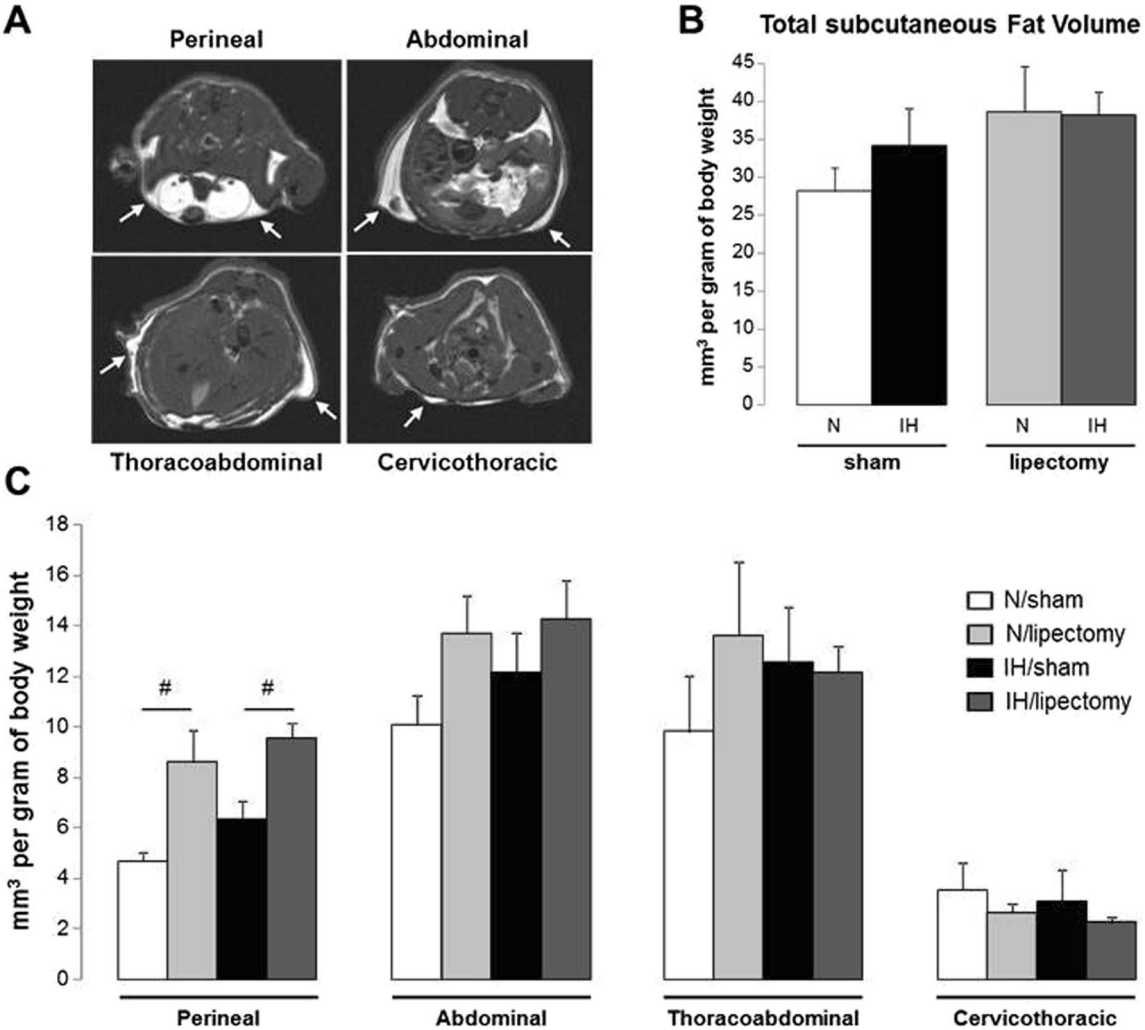
MRI quantification of subcutaneous white adipose tissue volume in mice exposed to intermittent hypoxia or normoxia. (**A**) Representative transverse MRI slices (arrow showing the subcutaneous fat), (**B**) total and (**C**) regional subcutaneous fat volume in the 4 body regions after 6 weeks of intermittent hypoxia (IH) or air (N) in lipectomized or sham-operated mice, n = 4 per group; *p < 0.05 N-sham *vs* N-lipectomy and IH-sham *vs* IH-lipectomy.

Compared to N-sham mice, IH-sham animals had lower body weight (Table 1 and Fig. 1A) but similar total white fat volume (Fig. 1B). The analysis of fat compartments showed that IH induces a 2-fold reduction of epididymal fat pads (Fig. 2A,B) whereas the other fat depots (mesenteric and subcutaneous) consistently showed some increase without reaching statistical significance (Figs 3B,C and 4B,C).

**Table 1:** Weight and glucose regulation in mice exposed to intermittent hypoxia or normoxia.

	SHAM		p N vs IH	LIPECTOMY		p N vs IH
	N	IH		N	IH	
n	7	7		6		6
Body weight after IH exposure (g)	27.4 ± 0.9	25.4 ± 0.4	<0.05	26.9 ± 0.7	26.0 ± 0.5	n.s.
**Insulin sensitivity**						
Blood glucose after 5 h of fasting (*mg/dl*)	199.8 ± 7.6	191.6 ± 10.0	n.s.	200.8 ± 9.7	192.6 ± 9.0	n.s.
Blood glucose AUC (*min*.*mg/dl*)	−3553 ± 578	−2146 ± 446	0.07	−3063 ± 362	−2907 ± 315	n.s.
Blood glucose nadir (*mg/dl*)	−85.6 ± 13.4	−54.7 ± 9.6	0.08	−77.2 ± 9.4	−75.2 ± 5.1	n.s.
**Insulin secretion**						
***Baseline measurements after 10 h of fasting***						
Blood glucose (*mg/dl*)	102.5 ± 8.8	100.0 ± 9.0	n.s.	95.5 ± 6.1	92.5 ± 8.2	n.s.
Plasma insulin (*ng/ml*)	0.159 ± 0.053	0.138 ± 0.035	n.s.	0.131 ± 0.023	0.122 ± 0.012	n.s.
***Measurements 15 min after glucose load***						
Blood glucose (*mg/dl*)	356.8 ± 11.5	344.0 ± 12.1	n.s.	387.8 ± 24.6	401.7 ± 27.5	n.s.
Plasma insulin (*ng/ml*)	0.173 ± 0.016	0.289 ± 0.032	<0.05	0.207 ± 0.026	0.219 ± 0.026	n.s.
Insulin secretion index (*a*.*u*.)	0.23 ± 0.02	0.63 ± 0.14	<0.05	0.34 ± 0.11	0.37 ± 0.09	n.s.

Insulin sensitivity and secretion were assessed at the fifth week of exposure to intermittent hypoxia (IH) or air (N) in lipectomized and sham-operated C57BL6 mice. AUC = area under the curve.

### Epididymal lipectomy induces alterations of body fat distribution

**Figure 5:**
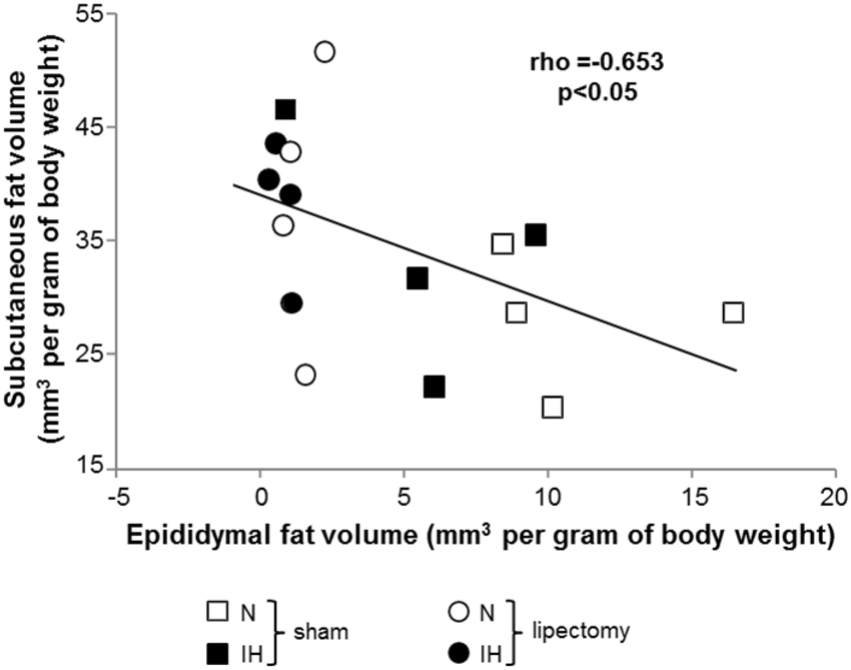
Correlation between epididymal and subcutaneous fat volumes in mice exposed to intermittent hypoxia or normoxia. Negative correlation between modification of epididymal and subcutaneous fat volume after 6 weeks of intermittent hypoxia (IH) or air (N) in lipectomized and sham-operated C57BL6 mice, n = 4 per group. Regression was calculated in the 4 pooled groups.

Normoxic and IH-lipectomized mice were not different for body weight (Table 1) and white fat volumes whatever the fat compartment (Figs 1B, 2B, 3B,C and 4B,C). Compared to sham animals, both normoxic and IH-lipectomized mice had similar total white fat volume but almost no epididymal fat pads even 6 weeks after surgery (Fig. 2A,B), and larger subcutaneous fat volume, in particular in the perineal compartment (Fig. 4C). Overall, epididymal and subcutaneous fat volumes were negatively correlated (rho −0.65, p = 0.011) (Fig. 5).

### IH impairs insulin sensitivity and release

At baseline, normoxic and IH-sham mice were not different for blood glucose whatever the duration of fasting (5 or 10 h), and for plasma insulin levels (Table 1). In contrast, dynamic tests exhibited insulin resistance in IH animals as shown by the trend to a lower blood glucose decrease after insulin challenge (i.e. smaller blood glucose AUC and nadir). After glucose challenge, IH animals had similar blood glucose levels but their insulin release and secretion index was significantly higher compared to normoxic animals. Overall our results showed an impaired insulin sensitivity and secretion after 6 weeks of IH in sham C57BL6 mice.

### Epididymal lipectomy prevents IH-induced metabolic alterations

IH-lipectomized mice were not different from normoxic mice regarding body weight, baseline blood glucose and insulin, insulin response (blood glucose AUC and nadir) and first-phase insulin secretion (Table 1). Compared to sham surgery, lipectomy prevented the IH-related disturbances in glucose homeostasis, as shown by the similar responses to glucose and insulin administration in both normoxic and hypoxic lipectomized mice.

## Discussion

The pathophysiology of OSA-induced metabolic alterations remains poorly understood. In the current study, we showed in non-obese C57BL6 mice that 6 weeks of IH resulted in alterations of body fat distribution (decrease in epididymal fat pads with non-significant increase in subcutaneous and mesenteric depots) and glucose dysregulation. Epididymal lipectomy before IH exposure led to an increase in subcutaneous fat volume and prevented IH-induced metabolic alterations.

### IH and lipectomy induce differential topographic changes of AT compartments

Literature is poor regarding the effects of IH *per se* on adipose tissue. Recent works showed that IH leads to various changes in epididymal AT such as adipocyte gene expression modifications^14, 15^, alteration of lipoprotein clearance pathways^12, 13^, sustained continuous hypoxia despite the normoxia-hypoxia cycles^16^ and inflammation^9–11^. In the present study we showed that in addition to functional and structural alterations, AT underwent specific topographic changes. Thanks to a MRI approach, a complete quantification of different AT compartments was realized. The four following fat territories were also assessed for their volume modifications after IH exposure: epididymal AT, subcutaneous AT, mesenteric AT and retroperitoneal AT. MRI quantification shows that the total fat mass was not modified in the hypoxic mice despite a significant loss of epididymal AT. Unlike epididymal AT that was clearly reduced in IH animals, subcutaneous and mesenteric fat volumes showed consistently higher values in the IH group, although these increases did not reach significance. A recent experimental study shows that IH increased Angptl4 only in epididymal AT probably due to a more severe hypoxia and activation of HIF1α in this AT territory^13^. As suggested in this study, the effects of IH could be tissue-dependent, explaining the differential response of AT compartments to IH regarding their volume changes. Furthermore the negative correlation between epididymal AT volume and subcutaneous AT volume that we observed confirms the compensatory mechanism following the epididymal adipose compartment reduction.

We showed that IH-induced insulin sensitivity impairment was associated with an epididymal AT wasting. At first sight, this phenomenon could seem paradoxical, as body weight and abdominal fat loss is thought to be beneficial for insulin sensitivity. However, using the same model of IH, we previously described an epididymal AT reduction in ApoE^−/−^ mice which was associated with adipocyte hypotrophy, increased plasmatic level of free fatty acids (FFAs) and insulin resistance^9^. These results are in agreement with a study from the group of Polotsky, showing an increased HOMA index and a decreased epididymal fat weight in lean mice exposed to 4 weeks IH^17^. Collectively, these data may suggest that IH increases epididymal AT lipolysis which can contribute to insulin resistance. Among mechanisms that could explain IH-induced lipolysis, IH activates the sympathetic nervous system^18^, which in turn is known to induce lipolysis through activation of the hormone-sensitive lipase in adipose tissue^19^. In 2010, Barcelo *et al*. showed that FFAs were significantly associated with apnoea/hypopnoea index, in a multiple regression model, after adjustment for age, sex, BMI and the presence of metabolic syndrome^20^. This study confirms the association between IH and FFAs secretion. Furthermore, it has been described that human abdominal visceral adipocytes are more sensitive to catecholamines-induced lipolysis than subcutaneous abdominal adipose cells^7^. These clinical data support the hypothesis of tissue-dependent effects of IH, in particular at adipose territories level.

To assess the implication of epididymal AT territory in the IH-induced metabolic alterations, we carried out a surgery to remove epididymal white fat. After 6 weeks of IH exposure, epididymal territory was still absent in both normoxic and hypoxic lipectomized mice. The total AT volume was similar in sham-operated compared to the lipectomized mice suggesting a compensation by other adipose tissues. After lipectomy, we observed a significant increase in subcutaneous AT volume at perineal level, in both normoxic and hypoxic mice, which was consistent with previous experimental results^21^. As observed in sham-operated mice after IH exposure, the imbalance between the different fat compartments following partial lipectomy is compensated by redistribution to the multi-depot “adipose organ”. This is consistent with the Kennedy’s “lipostatic” hypothesis that the “adipose organ” is able to adapt to its homeostasis modifications, in part through sensory innervations control and/or regulation of circulating humoral factors^22^.

### IH induces glucose homeostasis alterations

In the present study, we used non-obese C57BL6 mice. After IH exposure, basal blood glucose and insulin levels remained unchanged in the different groups. However during dynamic tests, animals exposed to 6 weeks IH showed an impaired glucose regulation (trend to lower glucose AUC and nadir after insulin challenge; similar blood glucose despite a higher insulin secretion after glucose challenge). Together, these results demonstrate that IH *per se* can induce metabolic disorders in mice (including impaired insulin sensitivity and secretion), independently of any obesity.

These results are in agreement with clinical studies in OSA patients showing a progressive worsening of insulin resistance and metabolic syndrome with OSA severity, independently of obesity^23, 24^. Previous studies also showed decreased insulin sensitivity in healthy volunteers^25^, lean mice^9, 10, 26, 27^ and cultured adipocytes^28^ exposed to IH. Very recently, we confirmed from cell culture, murine and human study that IH contributes independently to insulin resistance and we provided new evidence for the contributing role of IH-induced morphological and inflammatory remodeling of visceral AT in this IH-associated insulin resistance^11^. Taken together, these data suggests that, more than visceral AT volume, visceral AT morphological and inflammatory remodeling could be the main determinant of metabolic alterations (i.e. insulin resistance).

### Epididymal lipectomy leads to beneficial effects on IH-induced metabolic disturbances

As described above, IH induced epididymal AT alterations such as inflammatory phenotype acquisition or local sustained hypoxia. Local AT hypoxia as observed in obesity has been described to participate to the obesity-associated inflammation of AT^29^ and insulin resistance^30^. In the present study we demonstrated that insulin sensitivity was improved in hypoxic lipectomized animals compared to sham-operated hypoxic mice, consistent with previous study showing that removing visceral fat could prevent insulin resistance in obese aging rats^31^. The beneficial effect of lipectomy could be explained not only by the absence of inflammatory epididymal AT, but also by the compensatory extent of subcutaneous fat. Clinical studies have reported differences between visceral and subcutaneous AT regarding their implication in insulin sensitivity. Visceral adipocytes are defined by a peculiar metabolic profile compared to subcutaneous cells, characterized by hyperlipolytic activity and resistance to the antilypolytic effect of insulin^7^. McLaughin reported a positive association between visceral AT and insulin resistance, and between subcutaneous AT and insulin sensitivity, after BMI adjustment^32^. Despite some controversial results, the relationship between adipose tissue distribution and insulin resistance in human obesity is largely described in the literature^7^. Animal studies also reported an impact of adipose tissue distribution on insulin resistance. Subcutaneous lipectomy resulted in a compensatory increased in visceral AT accumulation and insulin resistance^33, 34^ and, conversely, it has also been shown that subcutaneous AT transplantation attenuated metabolic dysregulation in diet-induced obese mice^35^. Together these results show the important role of AT distribution in insulin resistance and that AT sensitivity to insulin is linked to its localization. Thus, in our present study, AT redistribution to a more insulin-sensitive tissue could also have participated in the beneficial effects of epididymal lipectomy on IH-induced insulin resistance.

### Limitations of the study and perspectives

OSA syndrome is a multicomponent and heterogeneous disease in terms of severity (number and type of respiratory events, hypoxia length and depth) and consequences according to individual susceptibility^2^. In this study, we used a IH stimulus that is well-described to mimic severe sleep apnea (cyclic 21–5% FiO_2_, 60 events per hour, nadir arterial oxygen saturation around 60%). However, would the metabolic consequences be the same with milder IH? The question is still open and it would be of interest to assess different magnitudes and frequencies of IH, as recent literature suggests that the IH-related consequences can be rather different and even beneficial depending on the severity of the hypoxic stimulus^36^. Whereas some studies reported beneficial effects of chronic hypobaric hypoxia (that mimics high altitude exposure) on glucose regulation^37, 38^, others demonstrated that both sustained and intermittent hypoxia impairs glucose regulation^16, 39^, with a more deleterious impact of IH on HOMA index^39^, oxidative stress and adipose tissue inflammation^16^. Finally, besides the IH stimulus, the subject vulnerability (i.e. obesity, aging, sedentarity, etc…) can also modify the resulting effects (either protective or detrimental) induced by a specific hypoxic stimulus. Thus, further dose-dependence studies comparing different magnitudes and frequencies of IH are needed to bring out physiological or molecular biomarkers that could predict whether IH would be beneficial or deleterious in OSA patients.

## Conclusion

In this study, IH led to a reduction in epididymal AT, and despite this reduction in epididymal fat, IH induced glucose dysregulation. This suggests that, rather than epididymal AT volume, qualitative alterations such as inflammatory remodeling could represent the main determinant of metabolic dysfunction. This deterioration in glucose regulation was prevented in epididymal-lipectomized mice, possibly through the prevention of the IH-induced epididymal AT alterations and the compensatory increase in subcutaneous AT.

## Methods

### Animals

Twenty six eight-week-old male C57BL6 mice fed on a standard-chow diet were used. The study was conducted in accordance with the European Convention for the Protection of Vertebrate Animals used for Experimental and Other Scientific Purposes (Council of Europe, European Treaties ETS 123, Strasbourg, 18 March 1986), and to the Guide for Care and Use of Laboratory Animals (NIH Publication No. 85–23, revised 1996). The protocol was approved by our local ethic comity (Grenoble Alpes University, 62-UHTA-HP2-MD-02, January 06, 2011).

### Intermittent hypoxia

IH was performed in experimental cages, as previously described^9^. The animals were exposed during daytime (n = 10 per cage, 8 h/day, cyclic 21–5% FiO2, 60 s cycle (60 events/h), lowest blood oxygen saturation up to 60%) for 6 weeks. FiO_2_ was measured with a gas analyzer (ML206, ADInstruments) throughout the experiment. Control animals (normoxic mice, N) were exposed to air in similar cages to reproduce similar noise and turbulences to those of the IH stimulus. Ambient temperature was maintained at 20–22 °C.

### Epididymal lipectomy

As previously published^9^, animals were either lipectomized or sham-operated under intraperitoneal ketamine-xylazine anesthesia (100 mg/kg-10 mg/kg). Bilateral epididymal WAT was carefully removed to keep the gonads intact. Sham animals underwent the same surgical protocol without removing the fat. Surgery sites were closed with 4–0 nylon absorbable monofilament (Ethicon, Inc.) and mice received buprenorphine (0.1 mg/kg, intraperitoneal). After 3 days of recovery, animals were exposed either to IH or N for 6 weeks, constituting four experimental groups (lipectomized IH and N mice, sham IH and N mice, n = 6–7 per group).

### Metabolic alterations

Insulin sensitivity and secretion were successively assessed with a 5-day interval at the sixth week of IH exposure using the insulin tolerance test and the insulin secretion index.

#### Intraperitoneal insulin tolerance test (IpITT)

Mice were fasted for 5 hours then weighed before blood was collected from the tail tip for baseline glucose determination (t = 0) using the OneTouch® Ultra® glucometer. Insulin (0.5 IU/kg body weight, Novo Nordisk A/S) was injected intraperitoneally, followed by further blood glucose measurements at 15, 30 and 60 minutes after the injection. Over the 60-minute period, the glucose nadir (the lowest blood glucose level) was calculated, and the glucose area under the curve (AUC) was measured using trapezoidal integration.

#### Insulin secretion index calculation

Mice were fasted for 10 hours then weighed. Blood was collected as described above for glucose and insulin measurements before (t0) and 15 minutes (t15) after an intraperitoneal glucose administration (2 g/kg body weight). Pancreatic insulin reserves consist of two pools of insulin. The first pool is rapidly mobilized (about 15 minutes after hyperglycemia) to quickly buffer the hyperglycemia. The second pool is a newly formed insulin, which occurs later and longer. The determination of plasma insulin 15 minutes after the glucose load reflects the immediate response of the pancreas to hyperglycemia (first-phase insulin secretion). Insulin levels were measured from 10 µl plasma samples using a Rat/Mouse ELISA kit (Millipore) according to the manufacturer’s instructions and expressed in ng/ml. The insulin secretion index was calculated ((insulin t15 − insulin t0)/(glucose t15 − glucose t0)) and expressed in arbitrary units^40^.

### Magnetic resonance imaging (MRI)

The analysis was performed the day following the end of IH exposure. Animals (n = 4 per group) were euthanized (intraperitoneal sodium pentobarbital, 50 mg/kg) to avoid respiratory motion artifacts, and imaged on a 7.0 T MRI system (Avance III console; Bruker, Germany; Grenoble MRI facility IRMaGe). Spin-echo T1-weighted images (TR/TE 500/8 ms; field of view 3.84 × 3.84 cm; slice thickness 1 mm; interslice gap 1 mm) were obtained through the entire mouse, from frontal to dorsal side (15 coronal slices), and from tail to head (64 transverse slices) (Fig. 1A).

The volumes of the four fat compartments (epididymal, subcutaneous, mesenteric and retroperitonal) were measured on the transverse slices by manual thresholding using the ImageJ software (version 1.45 s, NIH, USA). The volume of fat (mm3) was obtained by multiplying the area of fat of each slice (mm2) times 2 (1-mm slice thickness +1-mm interslice gap) and adding the volumes of each slice, then adjusted for 1 g of body weight. The total fat volume was calculated by summing the four compartments. The volume and topographic distribution of fat compartments was also assessed according to four body regions (cervicothoracic, thoracoabdominal, abdominal and perineal regions) (Figs 1A, 2A, 3A and 4A).

### Statistical analysis

Results were expressed as means ± SEM. Normality and variance homogeneity were tested and data were subsequently analyzed either by the non-parametric Kruskal-Wallis and Mann-Withney U tests, or a two-way analysis of variance, with IH and lipectomy as the independent variables. Subsequent multiple comparisons were made with the Bonferroni post-hoc test. Correlation between subcutaneous and epididymal fat volumes was evaluated by the non-parametric Spearman rank correlation test. Statistical significance was set at p < 0.05.
